# The Analgesic Efficacy of Transverse Abdominis Plane Block versus Epidural Block after Caesarean Delivery: Which One Is Effective? TAP Block? Epidural Block?

**DOI:** 10.1155/2018/3562701

**Published:** 2018-10-17

**Authors:** Ebru Canakci, Ahmet Gultekin, Zubeyir Cebeci, Bulent Hanedan, Anil Kilinc

**Affiliations:** ^1^Ordu University, School of Medicine, Training and Research Hospital, Department of Anesthesia and Reanimation, Ordu, Turkey; ^2^Golkoy State Hospital, Golkoy, Ordu, Turkey

## Abstract

**Introduction and Objective:**

TAP block has gained popularity to provide postoperative analgesia after abdominal surgery but its advantage over epidural analgesia is disputed. For lower abdominal surgeries, epidural analgesia has been the gold standard and time-tested technique for providing postoperative analgesia, but contraindications for the same would warrant need for other equally good analgesic techniques. The objective of this study is to compare the analgesic efficacy of both the techniques.

**Materials and Methods:**

Eighty patients in the ASA I-II risk group, undergone an elective C-section, were randomly assigned to the study. In the TAP group, before the C-section, a single-dose spinal anaesthesia was performed by administering 3 ml of 0.5% hyperbaric bupivacaine to the patients when they were in the sitting position. After the C-section, an ultrasound-guided bilateral TAP block was performed in these patients in the recovery room for postoperative analgesia. In the single-dose EPI group, the patients received 16 cc of isobaric bupivacaine, 3 mg of morphine, and 50 mcg of fentanyl, making a total volume of 20 cc and being administered to the epidural space.

**Results:**

A higher level of patient satisfaction was observed in the EPI group (*p*=0.003). The amount (mg) of total analgesics received by the patients in the first 24 hours of the postoperative period was statistically significantly higher (*p*=0.021) in the TAP group compared to the EPI group. The visual analogue scale (VAS) scores of the EPI group were significantly lower compared to that of the TAP group (*p* < 0.001).

**Conclusion:**

The epidural anaesthesia is still the golden standard to achieve a postcaesarean analgesia. Epidural anaesthesia is a considerably effective method in controlling the postoperative pain. We are of the opinion that epidural anaesthesia should be preferred in the first place to achieve a successful postcaesarean analgesia as it provides more effective pain control.

## 1. Introduction and Objective

For many years, epidural and caudal analgesia have been considered the gold-standard techniques after abdominal surgery for adults and children, respectively [1]. For this reason, new methods of regional anaesthesia have gained a widespread use recently [2]. Anaesthesiologists play an important role in postoperative pain management. For analgesia after lower abdominal surgery, epidural analgesia and ultrasound-guided transversus abdominis plane (TAP) block are suitable options. The study aims to compare the analgesic efficacy of both [3].

Epidural anaesthesia is preferred by most clinicians because, compared to spinal anaesthesia, it not only allows for a better control of the sensory level but also allows to perform a postoperative analgesia [4]. For lower abdominal surgeries, epidural analgesia has been the gold standard and time-tested technique for providing postoperative analgesia, but contraindications for the same would warrant need for other equally good analgesic techniques [3]. In recent years, the TAP block has gained popularity as an effective pain relief technique in patients undergoing a variety of abdominal operations. An increasing number of randomized-controlled trials and case reports in the literature have highlighted the analgesic effectiveness of the TAP block and proposed it as an alternative pain management technique in patients with contraindications to the use of opioids and/or neuraxial anaesthesia. Indeed, the TAP block avoids the risk of neuraxial complications and opioid complications in all patients [5]. TAP block the neural afferents of the abdominal anterior wall after spreading of the local anaesthetic agent in the neurofascial plan between the internal oblique and transversus abdominis muscle [6]. As the technique is relatively easy and its associated complications are minimal, the ultrasound-guided (US-guided) TAP block is commonly used today.

If analgesia management is performed properly, it will be associated with lower morbidity and mortality rates because severe pain can lead to cardiac arrhythmias, hypertension, and myocardial ischemia. In regard to performing postoperative analgesia in lower abdominal surgeries, peripheral nerve block methods are used including TAP block and epidural anaesthesia, which are accepted as the gold standard [7–9].

The objective of this study is to compare the efficiency of the bilateral TAP block and the single-dose epidural block in the postoperative analgesia management of 80 cases in the ASA I-II risk group who underwent C-section under elective conditions.

## 2. Materials and Methods

**Figure 1:**
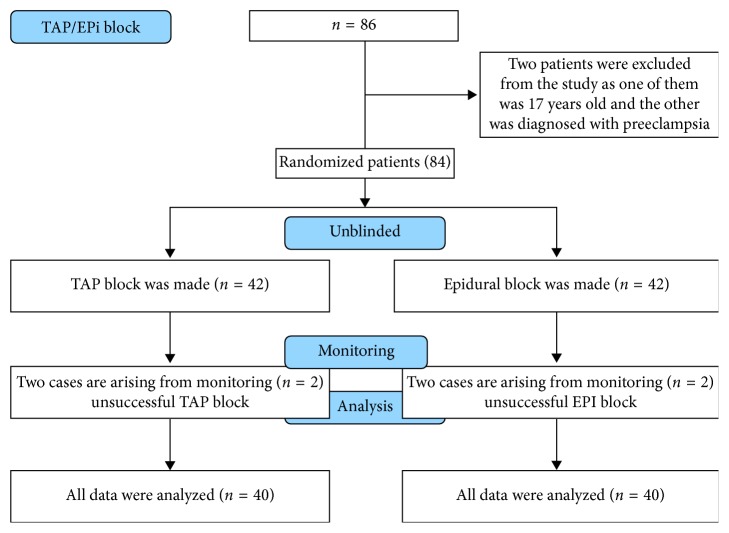
Consort flow diagram.

Ethical committee approval was obtained from the Ethics Committee of Clinical Investigations of Ordu University Medical Faculty (Decision no: 2017/22). Our study was conducted in Ordu University Faculty of Medicine, in the obstetric operation room, between May 2017 and December 2017. A written and signed informed consent was obtained from the patients who participated in this study. Detailed information about the regional anaesthesia techniques to be employed in this study was given to the patients. Of these two methods of anaesthesia and analgesia, the method to be administered was decided according to the preference of individual patients. Eighty patients in the ASA I-II risk group, aged between 18 and 50 years and who are at the appropriate gestational week to undergo an elective C-section, were assigned to either of the two study groups randomly and in an unblinded fashion. The patients were excluded from the study if they are under the age of 18 or older than 50 years, if they are in ASA III or ASA IV risk groups, if they are in the preterm period earlier than 38 weeks of gestation, if a foetal anomaly was detected during antenatal controls, if the following diagnoses were present including eclampsia, any gestational diseases such as HELLP syndrome and coagulopathies, if there was an infection in the region where the block would be performed, if the patients did not consent voluntarily or if they refused to participate in the study, and if they preferred general anaesthesia and rejected regional anaesthesia techniques. Based on an earlier study taken as a reference for the power analysis of our study, the sample size was determined at *α*=0.05 with a 90% power [7]. We included 40 patients in each group, which are, namely, the TAP group and the single-dose EPI group. Our study was designed as a randomized and unblinded prospective study. The study flow is presented in the Consort flow diagram (Figure 1). All patients received a 500 ml saline solution infused intravenously in the obstetric department before the operation. None of the patients was premedicated. After the patients in both groups were taken to the operating room, they were monitored by standard methods (SpO2, heart rate, and noninvasive arterial pressure). In the TAP group, a single-dose of spinal anaesthesia was administered to achieve anaesthesia during the surgery. After performing the required treatment and covering procedures, these patients received a 3 ml of 0.5% hyperbaric bupivacaine (Marcaine® Spinal Heavy, 0.5% ampoule, Astra Zeneca, Turkey) through the L3-L4 or L4-L5 intervertebral space while they were in the sitting position. For postoperative analgesia, a bilateral TAP block was performed after the operation in the recovery room.

**Figure 2:**
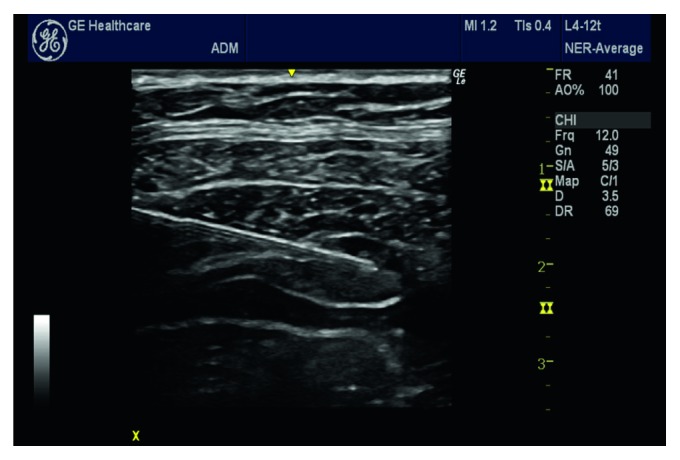
USG image of TAP block.

The lateral abdominal walls of the patients were sterilized with povidone iodine when the patients were in the supine position. The US equipment, GE HealthCareVenue40, USA, was used when performing the blocking procedure (Figure 2). A 12 MHz linear US probe coated with a sterile sheath was placed transversely on the unilateral abdominal wall between the costal margin and the iliac crest so that the practitioner stayed on the same side with that of the site of the procedure. To visualize the three lateral abdominal wall muscles, namely, the external oblique, internal oblique, and transversus abdominis muscle, the location of the probe was optimized by shifting it in the cephalocaudal or anterior-posterior directions or by shifting its angle. After the three abdominal muscles were clearly visualized, an 80 mm and 20-gauge needle (B. Braun® Stimuplex, Melsungen, Germany) was inserted from the anterior end of the probe by in-plane technique and proceeded forward. Viewing the position of the inserted needle guided by the ultrasound, 20 ml of 0.25% bupivacaine (Marcaine®, %0.5, Astra Zeneca, Turkey) solution was injected for the patients in the control group. The procedure performed on one side of the abdomen was repeated on the other side too.

In the single-dose epidural group (Group EPI), the epidural space was reached via the L3-L4 or L4-L5 intervertebral space using the hanging drop technique before the operation when the patients were in a sitting position. After making sure that there is no leakage of blood or cerebrospinal fluid, a 16 ml of 0.5% isobaric bupivacaine, 3 mg morphine (Morphine® HCL amp; 0.01 g, Galen İlaç, Turkey), and 50 mcg fentanyl (Talinat® 0.5 mg 10 ml amp, VEM İlaç, Turkey), making a volume of 20 cc in total, were injected into the epidural space. The catheter was not fixed on the skin. Surgery was performed in all patients when the sensory block reached the level of T4 in all cases. The sensorial block level was evaluated with a caloric response. All patients received 4 l/min oxygen with a mask during the operation.

Demographic data (age, weight, height, and parity) were recorded for each case. The moment of surgical incision was accepted as minute 0 and in every 0, 5, 10, 20, and 40 minutes, the mean arterial pressure (MAP) and the heart rate (HR) were recorded for each case. A 20% decrease in the blood pressure or a measurement of a 90 mmHg of systolic arterial pressure was defined as hypotension. An intravenous administration of 5 mg/ml of ephedrine was planned to be given to the patients when required. The total amount of ephedrine (mg) administered during the operation, the total duration of the operation, and the APGAR scores of the newborns in the 5th and 10th minutes after delivery were recorded. An intravenous administration of 50 mg of dexketoprofen (Ketavel® 50 mg ampule, Deva İlaç, Turkey) was planned to be administered to the patients when analgesia was required after the operation. The total amount of dexketoprofen (mg) administered to the patients within the first 24 hours after the surgery was recorded. The emerging postoperative adverse effects, including postoperative nausea, vomiting, and urinary retention, were recorded for each individual patient. The visual analogue scale (VAS) scoring was explained to the patients. A 10-point horizontal line was used as a scale, on which the point on the left end indicated an absolute lack of pain (0 points) and the point on the right end indicated a level of unbearable pain (10 points). The patients were asked to mark the level of the experienced pain on this scale. The VAS score recorded in the recovery unit was accepted as the time 0, and the VAS scores of each patient were recorded five times, namely, on the postoperative 2nd, 6th, 12th, and 24th time points. The level of patient satisfaction was measured numerically by a Likert scale ranging from one to five, **1**: “not satisfied at all,” **2**: “slightly satisfied,” **3**: “moderately satisfied,” **4**: “very satisfied,” and **5**: “highly satisfied”.

## 3. Statistical Analysis

The statistical analyses were performed by means of SPSS 19.0 for Windows software (SPSS Inc., Chicago, IL, USA). First, the data (VAS scores, MAP, HR, maternal satisfaction) were analyzed to test the quality of variance and the normality assumptions using Levene's test and the Shapiro–Wilk test. The significance was accepted at the level of *p* < 0.05 for all of the tests. Then, the Mann–Whitney *U* test was performed to determine the differences between the two study groups in terms of the amount of ephedrine required, the time elapsed till the skin incision was made, the time elapsed till the newborn was delivered, the total duration of the operation, the Apgar scores of the newborns in the 5th and 10th minutes, the number and type of the postoperative adverse effects, the time of the first breastfeeding, and the level of patient satisfaction. The MAP and HR values, recorded in the previously defined time points (minute 0 before the operation and in the postoperative 5th, 10th, 20th, and 40th minute), were analyzed by means of one-way ANOVA with repeated measures. The VAS scores recorded in the previously defined time points (VAS0, VAS2, VAS6, VAS12, and VAS24) were analyzed by means of the Friedman test. If the *p*-value was less than 0.05, the results were considered to be statistically significant. The results were expressed as means with standard deviation, median, and IQR.

## 4. Results

The demographic characteristics of the patients (age, weight, height, and parity) were not significantly different between the groups (Table 1).

**Figure 3:**
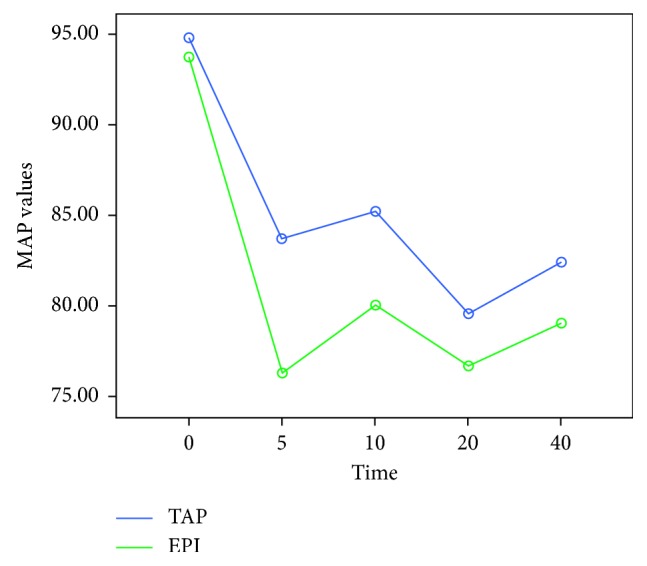
Comparison of MAP at different time points (0, 5, 10, 20, and 40 min) between the two groups.

Accepting the time point when the surgery was allowed as zero, the parameters measured in the 5th, 10th, 20th, and 40th minute, respectively, and the mean arterial pressure (MAP) values were significantly different between the two groups (*p* < 0.001). In the single-dose EPI group, the blood pressure of the patients showed a dramatical decline during the first five minutes (Figure 3).

**Figure 4:**
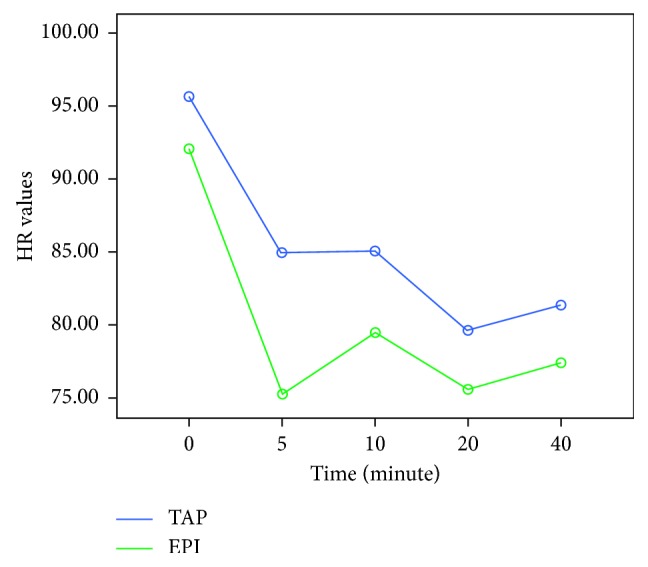
Comparison of the HR at different time points (0, 5, 10, 20, and 40 min) between the two groups.

The heart rate (HR) by the previously defined time points was significantly different between the two groups (*p* < 0.001). In the single dose EPI group, the heart rate showed a sharp decline in the 5th minute; however, the heart rate of the patients in the TAP group was more stable (Figure 4).

**Figure 5:**
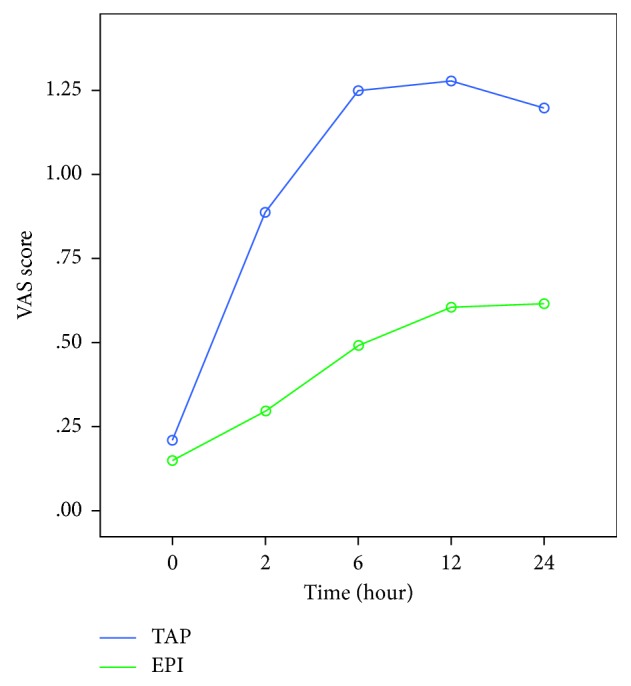
VAS scores of the groups.

The visual analogue scale (VAS) scores of the two groups were significantly different (*p* < 0.001). In the single-dose EPI group, the VAS scores were lower (Figure 5).

The perioperative amount of ephedrine given to the patients was similar between the study groups (*p*=0.086). The time elapsed till the skin incision was performed was found out to be similar in the TAP and the single-dose EPI groups. The total amount of the analgesic agent given to the patients in the first 24 hours after the surgery was statistically significantly higher in the TAP group compared to the single-dose EPI group (*p*=0.021). The time of the first requirement for an analgesic medication and the incidence of the postoperative side effects were not significantly different between the two groups. A significantly higher level of patient satisfaction was observed in the single-dose EPI group (*p*=0.003). The time elapsed till the first breastfeeding was found out to be shorter in the single-dose EPI group, but the difference was not statistically significant (*p*=0.15). The time elapsed till the first micturition and defecation in mothers were not significantly different between the two groups (*p*=0.222).

The results of our study demonstrated that the epidural anaesthesia remained to be the gold standard in achieving a postcaesarean analgesia because of relatively higher levels of patient satisfaction, lower postoperative VAS scores, and the lower amount of analgesic medications needed in the postoperative period.

## 5. Discussion

In our study, the level of the patient satisfaction was higher, and the VAS scores were lower in the single-dose EPI group. The total amount of analgesic medications given to the mothers in the first 24 hours after the operation was higher in the TAP group. These results demonstrate that epidural anaesthesia is considerably effective in achieving postoperative analgesia in the patients undergoing a C-section.

Breastfeeding is an action that requires the efforts of both the mother and the infant, and it is suggested that it should start as soon as possible after the birth. Efforts to investigate the factors associated with the breastfeeding issues after the birth deserve to receive any attention. The limitations of the previous studies on this subject have led to an uncertainty in the relationship between the regional anaesthesia and breastfeeding. In some studies, it was shown that epidural anaesthesia did not affect the postnatal milk release and a sufficient breastfeeding, whereas the study by Ashley et al. demonstrated that the duration and frequency of the breastfeeding were longer and higher, respectively, in the epidural anaesthesia group compared to the general anaesthesia group [8–12]. Our study found out that the first breastfeeding time in the single-dose EPI group was earlier compared to the TAP group, but the difference was not statistically significant. The reason for this moderate level of difference may be related to the higher level of patient satisfaction and lower postoperative VAS scores in the single-dose EPI group. We believe that both regional techniques have a positive effect on breastfeeding on the mothers' part. Our results are consistent with the results of Ashley et al.

In a placebo-controlled, double-blind study, Belavy et al. assigned 50 pregnant women in the appropriate gestational week in two groups, an experimental group, and a control group, to deliver mature infants with a C-section. Spinal anaesthesia was performed in both groups. After the operation, an US-guided TAP block was applied bilaterally to all patients in each group. The placebo control group received 40 mL of saline solution, and the other group received a 40 mL of 0.5% ropivacaine. The patient satisfaction was significantly higher the postoperative VAS scores were significantly lower in the ropivacaine group compared to the placebo group. The amount of morphine given to the patients was also found to be lower in the ropivacaine group [13]. The study by Belavy et al. demonstrates that an US-guided bilateral TAP block is an effective and efficient method of analgesia. Our results are consistent with the study of Belavy et al., in regard to the higher level of patient satisfaction, lower VAS scores, and total postoperative analgesic consumption in the single-dose EPI group. However, taking our clinical observations into consideration, we believe that the TAP block provides an effective analgesia after C-sections in the postoperative period.

A similar study to ours was conducted by Onishi et al. In this study, 94 pregnant women were divided into two groups before the elective C-section and all patients in both groups were given epidural anaesthesia. Based on the patient preferences, an US-guided bilateral TAP block was given to the 54 women after the C-section. The remaining 40 study participants, who refused to receive TAP blockade, were given 3 mg of morphine diluted with saline, administered to the epidural space. The authors reported that the amount of analgesics given to the patients were considerably low, and the level of patient satisfaction was remarkably high in performed both technique groups (epidurale analgesia group in addition to TAP block performed) [14]. They found a lower level of maternal satisfaction and higher VAS scores in the group treated with epidural morphine alone. However, Onishi et al. did not report the incidence of adverse events in their study. The study by Onishi et al. proves how efficient bilateral TAP block is in postcaesarean analgesia [14]. Unlike our study where we administered 3 mg morphine and 50 mcg fentanyl added to the 16 ml bupivacaine to the epidural space in the single-dose EPI group patients, all patients in Onishi et al.'s study received morphine to establish an epidural analgesia, and the volunteering patients were given an additional TAP block. We are not in favour of performing a TAP block when epidural catheters are available to administer analgesic medications. Although it is an improved technique guided with US, the risk of toxicity due to the local anaesthetic medications and the risk of infection due to the invasive nature of the process remain to be associated with the technique. Our study results are consistent with the results of Onishi et al. Because in our study, we also observed that epidural analgesia provided superior analgesia and maternal satisfaction than that of TAP block.

Fassoulaki et al. investigated the effect of anaesthesia on the duration of hospital stay and reported that the discharge from the hospital occurred earlier in the group of patients receiving regional anaesthesia [15]. Although Fassoulaki et al. compared the two groups of patients who were given either a general anaesthesia or a regional anaesthesia, in our study, there were no differences in regard to the time of the hospital discharge between the two regional anaesthesia groups. We think that this result is related to our institutional discharge policies rather than the anaesthesia method applied in our study. In our institution, the patients are discharged after 48 hours following the C-section if there are no emerging complications.

The opioids provide benefits when given through either the epidural or intrathecal routes; however, they are not free of the associated adverse effects. These side effects include nausea, vomiting, pruritus, urinary retention, and respiratory depression [16]. Dahlgren et al. demonstrated that opioids with adjuvant effects have reduced the incidences of postoperative nausea and vomiting, respectively [17]. Nausea and vomiting associated with C-section may occur due to several factors including the decreased cerebral blood flow during the operation leading to hypotension and the level of the blockade. In the latter, perioperative nausea and vomiting can occur due to the tension of peritoneal structures during the operation depending on the level of the blockade. It is commonly accepted that anaesthesia at the T4 level is adequate [16]. In all patients participating in the study, surgery was allowed after achieving a T4 sensory blockade. No antiemetic medications were given to the patients prophylactically. In our study, two patients in the single-dose EPI group (the group of patients receiving 3 mg morphine via the epidural route) and one patient in the TAP group reported nausea. None of the patients in the study developed urinary retention, vomiting, or pruritus. There was no significant difference between the two groups with regard to the time of the first micturition after the operation. Our results are consistent with the literature.

In conclusion, epidural anaesthesia is still the gold standard in postcaesarean analgesia. The lower postoperative VAS scores measured in all time points, the lower amount of analgesics given to the patients in the postoperative period, and the high levels of patient satisfaction in the single-dose epidural anaesthesia group demonstrate that epidural anaesthesia provides forceful and effective analgesia. Epidural anaesthesia is quite successful in the postoperative pain control; however, spinal anaesthesia is more commonly preferred by the anaesthetists as it is more practical. We believe that epidural anaesthesia should be preferred in the first place for a successful management of postcaesarean analgesia.

## Figures and Tables

**Table 1:** Demographic data of the groups.

Group	Group TAP	Group EPI
Age (year)	30.7 ± 4.1	29.6 ± 4.6
Weight (kg)	75.0 ± 12.9	78.1 ± 11.7
Height (cm)	145 ± 10.7	148 ± 12.4
Parity	2.9 ± 1.1	2.8 ± 1.2

## Data Availability

The data used to support the findings of this study are included within the article.
